# Fabrication of Composite Gel Electrolyte and F-Doping Carbon/Silica Anode from Electro-Spun P(VDF-HFP)/Silica Composite Nanofiber Film for Advanced Lithium-Ion Batteries

**DOI:** 10.3390/molecules28145304

**Published:** 2023-07-10

**Authors:** Caiyuan Liu, Xin Fang, Hui Peng, Yi Li, Yonggang Yang

**Affiliations:** Jiangsu Key Laboratory of Advanced Functional Polymer Design and Application, Department of Polymer Science and Engineering, College of Chemistry, Chemical Engineering and Materials Science, Soochow University, Suzhou 215123, China

**Keywords:** composite materials, electro-spun nanofiber, gel electrolyte, carbonaceous anode, lithium-ion batteries

## Abstract

The aim of this work is to effectively combine the advantages of polymer and ceramic nanoparticles and improve the comprehensive performance of lithium-ion batteries (LIBs) diaphragm. A flexible film composed of electro-spun P(VDF-HFP) nanofibers covered by a layer of mesoporous silica (P(VDF-HFP)@SiO_2_) was synthesized via a sol–gel transcription method, then used as a scaffold to absorb organic electrolyte to make gel a electrolyte membrane (P(VDF-HFP)@SiO_2_-GE) for LIBs. The P(VDF-HFP)@SiO_2_-GE presents high electrolyte uptake (~1000 wt%), thermal stability (up to ~350 °C), ionic conductivity (~2.6 mS cm^−1^ at room temperature), and excellent compatibility with an active Li metal anode. Meanwhile, F-doping carbon/silica composite nanofibers (F-C@SiO_2_) were also produced by carbonizing the P(VDF-HFP)@SiO_2_ film under Ar and used to make an electrode. The assembled F-C@SiO_2_|P(VDF-HFP)@SiO_2_-GE|Li half-cell showed long-cycle stability and a higher discharge specific capacity (340 mAh g^−1^) than F-C@SiO_2_|Celgard 2325|Li half-cell (175 mAh g^−1^) at a current density of 0.2 A g^−1^ after 300 cycles, indicating a new way for designing and fabricating safer high-performance LIBs.

## 1. Introduction

With the increasing demand for large-scale energy storage devices and portable electronic devices, energy storage components with a high energy density, such as lithium-ion batteries, zinc-ion batteries, zinc-air batteries, as well as supercapacitors, have been booming [1,2,3,4]. For lithium-ion batteries, besides the optimization of the composition and structure of electrode materials, many efforts have been exerted to enhance the performance of electrolyte/separator membranes [5,6]. Compared with commercial polyolefin separators, membranes made by poly(vinylidene fluoride) (PVDF) and its copolymers, especially, poly(vinylidene fluoride-co-hexafluoropropylene)(P(VDF-HFP)), showed superior safety property, shape flexibility, and scale ability because of their special chemical characteristics [7,8]. Moreover, ceramic fillers such as TiO_2_, SiO_2_, and ZnO have often been compounded with polymer membranes to improve their ionic conductivity, mechanical strength, and thermal stability [9,10]. However, the inhomogeneous dispersion of inorganic fillers in the polymer matrix results in a sharp decrease in the compatibility of the two phases, affecting the practicability of separator membranes [11]. Therefore, to improve the comprehensive properties of polymer/ceramic composite separators, technical enhancement and structural modification are necessary and feasible [12,13,14,15]. For example, Guo et al. [13] prepared a composite membrane by covering both sides of a commercial PP membrane with SiO_2_/P(VDF-HFP) composite coating. The thin 700 nm ceramic/polymer coating layer avoided obstructing ion conduction and significantly improved the thermal stability of the diaphragm. Wang et al. [14] reported an electro-spun PVDF-HFP composite nanofiber membrane. The three-dimensional network structure composed of nanofibers provides a large number of pores. In addition, proper doping of AgNWs@SiO_2_ and single-ion conducting polymer electrolyte could obtain a greater liquid electrolyte adsorption rate, hence improving the mechanical properties and thermal stability. Zhang et al. [15] prepared electro-spun PVDF-HFP nanofiber membranes doped with functionalized silica nanoparticles. The uniformly dispersed nano-particles in the polymer matrix effectively promote the mechanical and electrical properties of the composite electrolyte, and the introduction and fixing of anionic groups in the electrolysis of the gel polymer make the assembled symmetrical lithium batteries show a high lithium-ion migration number and excellent lithium plating/stripping performance.

Another way to enhance the comprehensive performance of secondary batteries is to improve the interfacial contact of the separator and solid electrode [16,17,18,19,20,21,22,23,24]. For example, Zhou et al. [21] reported that a 2 wt% LiF-coated garnet Li_6.5_La_3_Zr_1.5_Ta_0.5_O_12_ (LLZT) surface could effectively isolate air and water, thus greatly reducing the generation of the lithium-ion insulation layer and decreasing the interfacial resistance of composite LLZT nanoparticles/electrode. Shen et al. [22] prepared a new integrated separator/positive electrode by applying Zeolitic Imidazolate Framework-4 (ZIF-4)/PVDF slurry directly onto the Li[Ni_1/3_Co_1/3_Mn_1/3_]O_2_ positive electrode. The organic/inorganic hybrid composite diaphragm showed good thermal stability, a high absorption rate of liquid electrolyte, and low interfacial resistance between the electrode and the separator. Wang et al. [23] applied an in situ solid polymer layer directly onto the anode surface. The flexible conductive polymer layer doped with lithium salt acted as an excellent anode protection film, effectively improving the safety of the battery. More importantly, this solid polymer electrolyte closely bonded with the electrode, resulting in low interface impedance of less than 100 Ω. However, the abovementioned methods usually focus on the coating modification, ignoring the enhancement of the structural compatibility of the solid electrolyte/solid electrode.

In this work, we provided a facile way to fabricate a high-performance separator as well as improve interfacial contact between the solid separator and solid electrodes. A composite film composed of P(VDF-HFP) nanofibers covered by a layer of mesoporous silica (denoted as P(VDF-HFP)@SiO_2_) was fabricated through electrospinning followed by sol–gel preparation. It was then applied as a separator as well as scaffold to absorb organic electrolyte to make quasi-solid-state electrolyte (usually referring to gel electrolyte, GE) for LIBs. Meanwhile, F-doping carbon/silica composite nanofibers (F-C@SiO_2_) were also gained by carbonizing P(VDF-HFP)@SiO_2_ composite film. The electrochemical tests showed that the P(VDF-HFP)@SiO_2_ separator presented high electrolyte uptake, ionic conductivity, and excellent compatibility with active Li metal, and the F-C@SiO_2_ was a promising LIBs anode material. The assembled F-C@SiO_2_|P(VDF-HFP)@SiO_2_-GE|Li half-cell exhibited superior electrochemical performance as expected.

## 2. Results and Discussion

### 2.1. P(VDF-HFP)@SiO_2_ Composite Nanofiber Film

**Figure 1:**
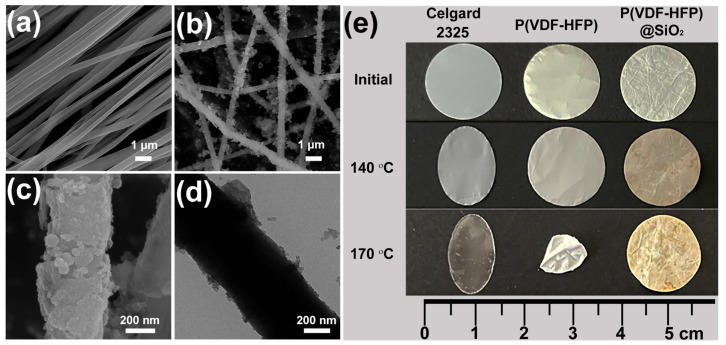
(**a**–**c**) FE-SEM, (**d**) TEM images of (**a**) P(VDF-HFP) and (**b**–**d**) P(VDF-HFP)@SiO_2_ composite nanofibers and (**e**) optical photos of corresponding shrinkage before and after heat treatment of Celgard 2325, P(VDF-HFP), and P(VDF-HFP)@SiO_2_ composite membrane.

The morphology and structure of the obtained electro-spun P(VDF-HFP) nanofibers and P(VDF-HFP)@SiO_2_ composite nanofibers are showed by their FE-SEM and TEM images (Figure 1a–d). The P(VDF-HFP) nanofibers with a diameter of about 350 nm have a smooth surface, uniform size, and are stacked together to make a three-dimensional network film. After sol–gel preparation, silica layers with an average thickness of 50 nm adhere to the surfaces of P(VDF-HFP) nanofibers tightly. TGA analysis (Figure S1a) discloses that the starting decomposition temperature of P(VDF-HFP)@SiO_2_ membrane is 350 °C, proving excellent thermal stability. The thermal dimensional stability of P(VDF-HFP)@SiO_2_ membrane was tested and compared with Celgard 2325 and P(VDF-HFP) membranes, as is displayed in Figure 1e. After being stored at 140 °C for 1 h, Celgard 2325 showed obvious thermal shrinkage behavior, while the size of P(VDF-HFP) and P(VDF-HFP)@SiO_2_ membranes changed only a little. At the temperature of 170 °C, Celgard 2325 shrank further, P(VDF-HFP) showed serious shrinkage, while P(VDF-HFP)@SiO_2_ changed only slightly. This result shows that the coating of the SiO_2_ layer can effectively reduce thermal shrinkage of the polymer diaphragm, thereby inhibiting the battery short circuit problem and improving the battery’s safety.

### 2.2. F-C@SiO_2_ Composite Nanofibers

**Figure 2:**
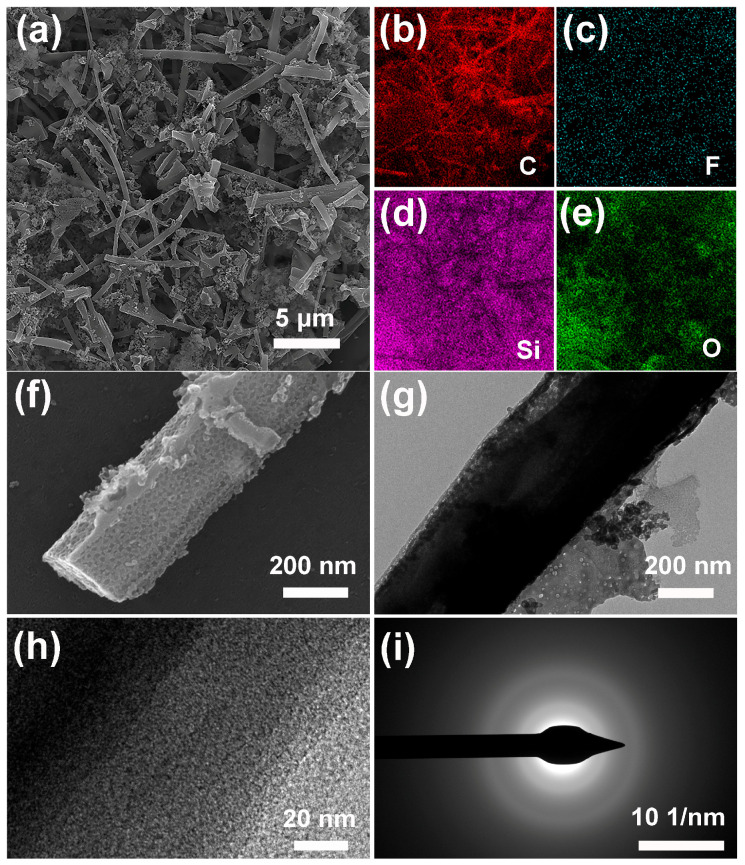
(**a**,**f**) FE-SEM, (**b**–**e**) EDS, (**g**) TEM, (**h**) HR-TEM images, and (**i**) SAED pattern of F-C@SiO_2_ composite nanofibers.

When the P(VDF-HFP)@SiO_2_ film was heated in nitrogen at 700 °C, F-C@SiO_2_ composite nanofibers were obtained and displayed in Figure 2. Long P(VDF-HFP) nanofibers carbonized and broke to form shorter carbonaceous nanofibers. The EDS result (Figure 2b–e) verifies the uniform distribution of C, F, Si, and O elements. The F-C@SiO_2_ composite comprises 63.3 wt% of F-doping carbon, as calculated from Figure S1b. The XPS results further disclose the chemical composition of F-C@SiO_2_ composite nanofibers (Figure S2). The full spectrum (Figure S2a) confirms the existence of four elements: C, O, Si, and F. In Figure S2b, the peaks at 284.6 eV, 285.0 eV, and 286.2 eV correspond to C-C, C-F, and C-F_3_, respectively. In Figure S2c, the peaks at 687.6 eV and 688.2 eV correspond to ionic F and semi-ionic F, respectively. In Figure S2d, the peaks at 104.4 eV correspond to Si-O. Uniformly distributed mesopores can be clearly observed on the surfaces of the fibers (Figure 2f). HR-TEM (Figure 2h) and SAED images (Figure 2i) indicate that no large crystalline zones exist in F-C@SiO_2_. In the WAXRD pattern (Figure S3a), a broad diffraction peak appears at 2*θ* = 21.5°, which belongs to the (002) crystal plane of graphite, indicating that the degree of crystallinity for carbon in the nanofibers is low. In the Raman spectrum (Figure S3b), D and G bands were observed at 1339 cm^−1^ and 1584 cm^−1^, respectively. The *I*_G_/*I*_D_ ratio calculated by Gaussian fitting is about 0.84, further confirming that the carbon in F-C@SiO_2_ is mainly amorphous. Nitrogen adsorption–desorption isotherms and BJH pore size distribution plots of P(VDF-HFP)@SiO_2_ and F-C@SiO_2_ composite nanofibers are showed in Figure S4. Two type IV curves with H3 hysteresis cycles confirm the existence of micro- and mesopores, with a specific surface area and pore volume of 99.7 m^2^ g^−1^/0.277 cm^3^ g^−1^ and 285.1 m^2^ g^−1^/0.453 cm^3^ g^−1^, respectively. The coarse fiber surface and plenty of micro- and mesopores within the composite nanofibers provide enough space for the holding of electrolyte and ensure the rapid transfer of lithium ions.

### 2.3. P(VDF-HFP)@SiO_2_-Based Gel Electrolyte

**Figure 3:**
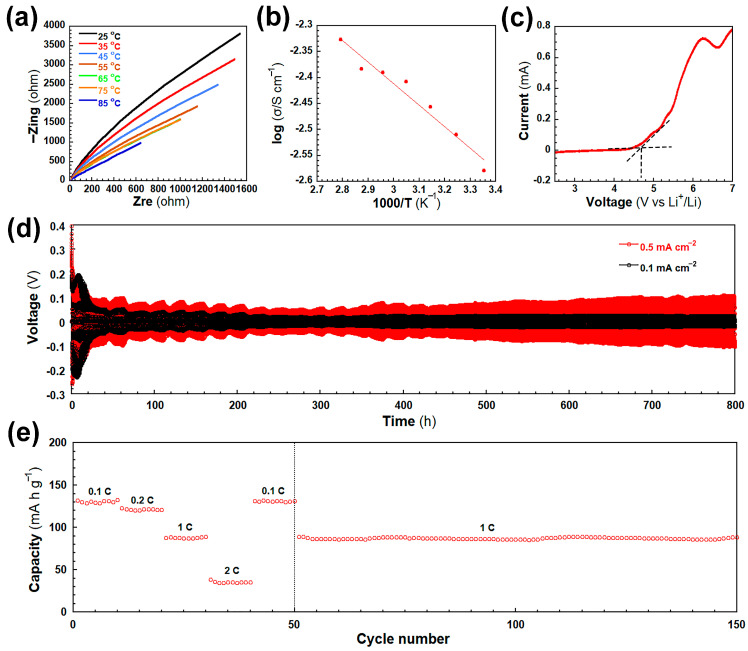
Electrochemical performance of P(VDF-HFP)@SiO_2_-GE membrane: (**a**) AC impedance spectra of at different temperatures; (**b**) ionic conductivity measured at different temperatures; (**c**) LSV curve; (**d**) voltage profiles for Li plating/striping experiment as a function of time at the current density of 0.1 and 0.5 mA cm^−2^; (**e**) rate performance at various rates and cycling performance at 1 C (1 C= 170 mA g^−1^).

When the P(VDF-HFP)@SiO_2_ film was cut into a 16 mm diameter wafer and used to soak organic electrolyte (1M LiPF6 in 1:1 EC/DMC) till saturation, the P(VDF-HFP)@SiO_2_-GE membrane was gained. The electrolyte uptake of the P(VDF-HFP)@SiO_2_ membrane was calculated based on the mass difference in the membranes before and after sufficient immersion in the liquid electrolyte according to the following formula,

(1) $$\mathrm{Uptake}\;(\%)=\frac{W_{w}-W_{d}}{W_{d}}\times \;100$$

where *W_d_* and *W_w_* are the mass of the membrane before and after immersion in the liquid electrolyte, respectively. The electrolyte uptake is 1000 wt%, much higher than the commercial celgard 2325 membrane (~90 wt%) and many reported electro-spun P(VDF-HFP)-based scaffolds (Table S1) [25,26,27,28]. The electrochemical properties of P(VDF-HFP)@SiO_2_-GE were tested and are displayed in Figure 3. The ionic conductivity (*σ*) of P(VDF-HFP)@SiO_2_-GE was investigated using AC impedance analysis on a stainless steel symmetrical cell (SS|P(VDF-HFP)@SiO_2_-GE|SS), which can be further calculated by the formula,

(2) $$\sigma =\frac{l}{R_{b}S}$$

where *l* is the electrolyte membrane thickness, *R_b_* stands for the electrolyte bulk resistance measured by EIS, and *S* is the cross-sectional area. The value of *σ* increases with temperature due to a decrease in the ohmic resistance of the electrolyte, as is presented in Figure 3a,b. The activation energy of Li^+^ conduction can be calculated from Figure 3b by the Arrhenius equation:

(3) $$\sigma =A\;\exp(-\frac{E_{a}}{\mathrm{RT}})$$

where *A* is the pre-exponential factor, *E_a_* stands for the activation energy, *T* is the absolute temperature, and *R* stands for the Boltzmann constant. The activation energy of ion conduction is estimated to be 7.9 kJ mol^−1^. The ionic conductivity of P(VDF-HFP)@SiO_2_-GE at 25 °C is 2.6 mS cm^−1^, which meets the minimum operating limit of LIBs at room temperature (1 mS cm^−1^) [29,30]. As Figure 3c shows, the decomposition voltage of P(VDF-HFP)@SiO_2_-GE at room temperature is 4.6 V, larger than commercial LIBs (4.5 V vs. Li/Li^+^) [31], indicating that this nanofiber membrane possesses good electrochemical stability. The charge–discharge performance of the assembled Li|P(VDF-HFP)@SiO_2_-GE|Li cell was tested in the voltage range of 2.5–4.2V. A periodic constant current (0.1 and 0.5 mA cm^−2^) was applied to the cell, and the stripping/plating behavior of lithium ions on the electrode surface was investigated by recording voltage changes, as shown in Figure 3d. The voltage reduction at the beginning of the cycle derives from the polarization voltage formed by the concentration polarization inside the cell, and then the voltage remains relatively stable, suggesting the formation of stable interfaces and benign compatibility between the metallic Li foil and the P(VDF-HFP)@SiO_2_-GE membrane. The LiFePO_4_|P(VDF-HFP)@SiO_2_-GE|Li cell can work well at different rates (0.1–2 C), as is shown in Figure 3e. When the current density returns from 2 C to 0.1 C, the specific discharge capacity of the cell almost completely recovers, showing excellent rate performance. Subsequently, the same cell continues a long cycle test at 1 C and its discharge capacity recovers and gains no obvious capacity loss till 1000 cycles (Figure S5), showing excellent cycle stability. After 1000 stable cycles, the charge-transfer impedance of the cell reduces from 680 Ω to 220 Ω (Figure S6), further demonstrating improved interface contact conditions between the electrolyte and the electrode. Herein, the P(VDF-HFP)@SiO_2_-GE membrane exhibited superior electrochemical characteristics such as higher ionic conductivity and a better capacity retention ratio after long cycles at a high rate than many reported electro-spun P(VDF-HFP)-based gel electrolytes (Table S1), indicating that it could be a prospective candidate for practical application in LIBs.

### 2.4. F-C@SiO_2_|P(VDF-HFP)@SiO_2_-GE|Li Battery

**Figure 4:**
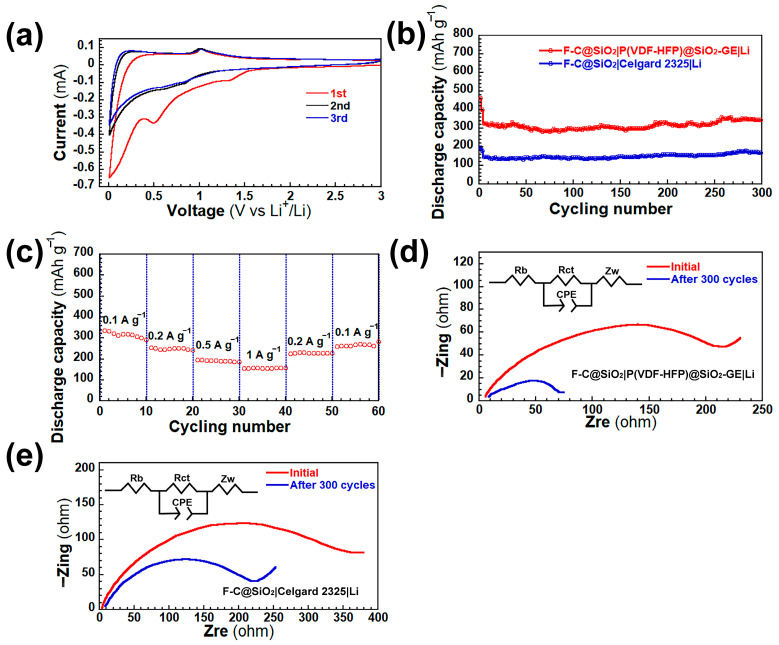
Electrochemical performance of F-C@SiO_2_|P(VDF-HFP)@SiO_2_-GE|Li half-cell: (**a**) cyclic voltammograms; (**b**) cycling performance at 0.2 A g^−1^; (**c**) rate performance at different current densities from 0.1 A g^−1^ to 2 A g^−1^; (**d**) nyquist plots in the frequency range from 10 Hz to 100 KHz before and after 300 cycles; (**e**) nyquist plots of F-C@SiO_2_|Celgard 2325|Li half-cell at the same testing condition.

Using F-C@SiO_2_ to make the working electrode, metallic Li foil as the counter electrode, and P(VDF-HFP)@SiO_2_-GE as the separator as well as quasi-solid-state electrolyte, a CR2016 coin-type half-cell (F-C@SiO_2_|P(VDF-HFP)@SiO_2_-GE|Li) was integrated. Its electrochemical behavior was tested and is shown in Figure 4. In the cyclic voltammogram (Figure 4a) measured at 0.01–3.0 V with a scan rate of 0.1 mV s^−1^, the cathodic peak at around 0.5 V is because of the decomposition of electrolyte and the formation of SEI film on the fiber surface [32]. The almost coincided second and third cyclic curves indicate good cyclic stability of the cell. The cycling performance of the F-C@SiO_2_ electrode at 0.2 A g^−1^ is shown in Figure 4b and Figure S7. The first charge and discharge capacities are 1514.2 and 465.1 mAh g^−1^, respectively. The irreversible capacity loss is related to the formation of the SEI film and other lithiation reactions. After 300 cycles, a stable discharge capacity of 340 mAh g^−1^ is gained. It is interesting to find that the capacity of the cell using P(VDF-HFP)@SiO_2_-GE is nearly twice of the cell using the commercial celgard 2325 separator, which is probably related to the larger electrolyte absorption of the P(VDF-HFP)@SiO_2_ membrane and better interfacial contact between the P(VDF-HFP)@SiO_2_-GE and F-C@SiO_2_ electrode [33,34]. The rate behavior of the F-C@SiO_2_|P(VDF-HFP)@SiO_2_-GE|Li cell at various current densities is displayed in Figure 4c. Capacities of 511.5, 355.6, 253.6, and 211.1 mAh g^−1^ at current densities of 0.1, 0.2, 0.5, and 1 A g^−1^ were achieved, respectively. When the current density returns to 0.2 and 0.1 A g^−1^, the recovery rate of the capacity is higher than 90%. In the Nyquist plot (Figure 4d,e), after 300 cycles, the interfacial impedance of the F-C@SiO_2_|P(VDF-HFP)@SiO_2_-GE|Li cell reduces from 255 Ω to 70 Ω, while that of the F-C@SiO_2_|Celgard 2325|Li cell reduces from 365 Ω to 224 Ω. P(VDF-HFP)@SiO_2_-GE shows better interfacial compatibility with the F-C@SiO_2_ electrode, which is due to their similar structure and composition. In addition, the sharp reduction in impedance suggests good electrical conductivity and a rapid charge transfer reaction, benefitting lithium-ion insertion/extraction.

Herein, the integrated F-C@SiO_2_|P(VDF-HFP)@SiO_2_-GE|Li cell exhibited superior electrochemical performance, which was reasonably ascribed to the deliberately designed composition and structure as well as the enhanced interfacial contact between the electrodes and gel electrolyte. As is known, the more similar the composition and structure of the two phases, the better the compatibility between them is. The mesoporous SiO_2_-coated nanofibers which exist both in the gel electrolyte and F-C@SiO_2_ electrode undoubtedly improve the interfacial compatibility between them. Meanwhile, plenty of the mesopores on the nanofibers greatly increase the electrolyte uptake, benefitting the interfacial contact of the gel electrolyte (separator) and the metallic Li electrode. The hard SiO_2_ layer also enhances the strength of the nanofiber membrane, lessening the risk of piercing through by Li dendrite. As far as we know, this is the first report on assembling an electro-spun nanofiber separator and its derivate carbonaceous electrode in one battery.

## 3. Experimental Section

### 3.1. Materials

Polyvinylidene-hexafluoropropylene (P(VDF-HFP), Mw = 455,000), and N,N-dimethy-l-formamide (DMF, >99.9%) derive from Aladdin. LiFePO_4_ was purchased from Shanghai D&B Biotechnology Co., Ltd. (Shanghai, China). Vinyl carbonate (EC) and dimethyl carbonate (DMC) were produced by Guotai Huarong Chemical New Materials Co., Ltd. (Suzhou, China). Cetyl trimethyl ammonium bromide (CTAB, >98%) was acquired from Bede Pharmaceuticals Ltd. (Harare, Zimbabwe). Tetraethyl orthosilicate (TEOS, >99.5%) and acetone derive from Jiangsu Qiangsheng Functional Chemical Co., Ltd. (Suzhou, China). Anhydrous ethanol (>99.7%) and ammonia (NH3·H2O, 25 wt%) were provided by Sinopharm Chemical Reagent Co., Ltd. (Shanghai, China). Celgard 2325 was bought from Suzhou Sukrain Instrument Co., Ltd. (Suzhou, China). All materials were used as received without further purification.

### 3.2. Synthesis of Electro-Spun P(VDF-HFP)/Mesoporous Silica Composite Nanofiber Film and F-Doped Carbon/Silica Composite Nanofibers

Electro-spun P(VDF-HFP) nanofiber film was synthesized according to previous work [35]: P(VDF-HFP) particles (20 wt%) were dissolved in DMF/acetone solution (*v*/*v* = 7:3) and stirred at 50 °C for 3 h to obtain uniform spinning solution. P(VDF-HFP) nanofibers were produced by electrospinning. Regarding the experimental conditions, we applied a positive voltage of 10 kV, negative voltage of −5 kV, collection distance of 15 cm, injection speed of 0.08 mm s^−1^, and moderated at 40%. The collected nanofibers were dried overnight in a 60 °C oven.

A total of 200 mg of CTAB was completely dissolved in 200 mL of EtOH/H_2_O solution (EtOH/H_2_O = 1/100, *v*/*v*) at 40 °C. Then, 50 mg of above-prepared P(VDF-HFP) electrospinning film was immersed in the solution and stood for 2 min. After that, 1 mL of TEOS followed by 0.1 mL of concentrated ammonia solution was added dropwise to the solution and it was left to stand at 40 °C for 8 h. The as-prepared composite film was washed with deionized water to remove the surface impurities, heated in ethanol with reflux for 12 h to remove CTAB, and dried at 70 °C for 24 h. Finally, P(VDF-HFP)@SiO_2_ composite nanofiber film was obtained.

**Scheme 1:**
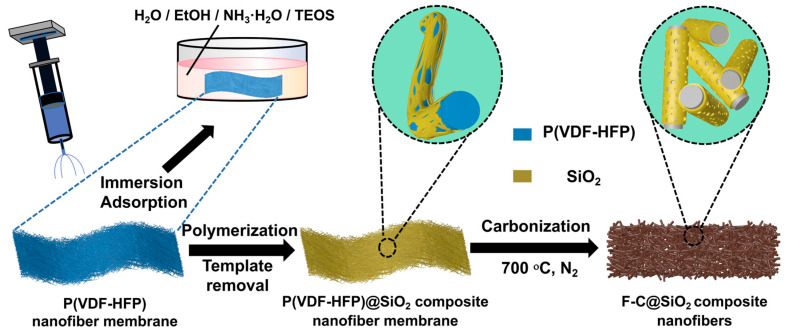
Illustration of the fabrication process for P(VDF-HFP)@SiO_2_ composite nanofiber membrane and F-C@SiO_2_ composite nanofibers.

The prepared P(VDF-HFP)@SiO_2_ composite nanofiber film was heated in nitrogen at 700 °C at a rate of 10.0 °C/min for 6 h. After naturally cooling to room temperature, F-doped carbon/silica composite nanofibers (designated as F-C@SiO_2_) were obtained. The fabrication process for P(VDF-HFP)@SiO_2_ composite nanofiber membrane and F-C@SiO_2_ composite nanofibers was illustrated in Scheme 1.

### 3.3. Assembly of LiFePO_4_/Li and F-C@SiO_2_/Li Batteries and Electrochemical Tests

For the preparation of the LiFePO_4_ electrode, LiFePO_4_ (80 wt%) powder, acetylene black (10 wt%), and PVDF (10 wt%) (total mass is 80 mg) were evenly dispersed in NMP and stirred at room temperature for 5 h. The mixture was scraped onto aluminum foil, dried overnight in a 70 °C oven, and cut into 14 mm diameter rounds. Using the LiFePO_4_ electrode as the positive electrode, lithium metal as the counter electrode, and P(VDF-HFP)@SiO_2_-GE as the separator, the LiFePO_4_/Li battery was assembled in a glovebox filled with argon gas.

For the preparation of the F-C@SiO_2_ electrode, F-C@SiO_2_ (80 wt%) powder, acetylene black (10 wt%), and PVDF (10 wt%) (total mass is 40 mg) were evenly dispersed in NMP and stirred at room temperature for 5 h. The mixture paste was scraped onto copper foil, dried overnight in a 70 °C oven, and cut into a 14 mm diameter wafer. Using the F-C@SiO_2_ electrode as the working electrode, lithium metal as the counter electrode, and P(VDF-HFP)@SiO_2_-GE and Celgard 2325 as the separators, the F-C@SiO_2_|P(VDF-HFP)@SiO_2_-GE|Li and F-C@SiO_2_|Celgard 2325|Li batteries were assembled in a glovebox filled with argon gas.

Electrochemical tests were conducted with an electrochemical workstation (CHI660E, CH Instruments, Austin, TX, USA), and galvanostatic charge/discharge tests were carried out with LAND systems. The electrochemical impedance was acquired in a frequency range from 0.01 Hz to 100 kHz at different temperatures with an AC amplitude of 5 mV. The electrochemical stability window was measured using linear sweep voltammetry (LSV). The potential was swept between open circuit voltage and 7.0 V (V vs. Li/Li^+^) with a scan rate of 5.0 mV s^−1^. The interfacial compatibility between the P(VDF-HFP)@SiO_2_-GE membrane and Li metal electrode was tested based on the charge/discharge cycling performance of the symmetrical Li|P(VDF-HFP)@SiO_2_-GE|Li battery with a Land CT2001A cell tester at room temperature.

### 3.4. General Methods

Field emission scanning electron microscopy (FE-SEM) was performed using a Hitachi 4800 instrument at 3.0 kV. Transmission electron microscopy (TEM) and high-resolution TEM (HR-TEM) images, as well as selective area electron diffractions (SAED), were obtained using an FEI TecnaiG220 at 200 kV. Wide angle X-ray diffraction (WAXRD) patterns were collected with an X’Pert-Pro MPD X-ray diffractometer using Cu Kα radiation (λ = 0.154 nm). Raman spectra were recorded using a Jobin Yvon Horiba HR 800 LabRAM confocal microprobe Raman system with Ar laser excitation (514.5 nm) and a power of 10 mW. Specific surface area and pore-size distribution were determined based on the Brunauer–Emmett–Teller (BET) and Barrett–Joyner–Halon (BJH) methods using N_2_ adsorption–desorption isotherm measured by a Micromeritics Tristar II 3020 instrument. Thermogravimetric analysis (TGA) was performed on a Thermal Analysis TG/DTA 6300 instrument with a heating rate of 10 °C min^−1^ under air from 25 °C to 800 °C. X-ray photoelectron spectroscopy (XPS) was performed on a Pass Energy 100.0 eV, Al K Alpha.

## 4. Conclusions

In conclusion, P(VDF-HFP)@SiO_2_ composite nanofiber film was synthesized via electrospinning followed by a sol–gel reaction. After carbonization, F-C@SiO_2_ composite nanofibers were gained. Electrochemical characterization disclosed that P(VDF-HFP)@SiO_2_-based gel electrolyte exhibited high ionic conductivity and thermal stability, while the F-C@SiO_2_-based anode showed good Li-storage capacity. Because of the enhanced interfacial contact between the electrodes and the gel electrolyte, the assembled F-C@SiO_2_|P(VDF-HFP)@SiO_2_-GE|Li cell exhibited superior electrochemical performance. On account of the economic raw materials, facile fabrication process, and good comprehensive properties, this work has demonstrated a new design for safe next-generation LIBs.

## Data Availability

The data presented in this study are available in the Supplementary Materials.

## Supplementary Materials

The following supporting information can be downloaded at: https://www.mdpi.com/article/10.3390/molecules28145304/s1, Figure S1: TGA curve of (a) P(VDF-HFP)@SiO_2_ and (b) F-C@SiO_2_. Figure S2. The XPS curve of F-C@SiO_2_ composite nanofibers: (a) full spectrum, (b) C 1s, (c) F 1s, (d) Si 2p orbital high-resolution spectrum. Figure S3: (a) WAXRD pattern and (b) Raman spectrum of F-C@SiO_2_. Figure S4: (a) Nitrogen sorption isotherms and (b) BJH pore size distribution plot calculated from the adsorption branch of P(VDF-HFP)@SiO_2_ and F-C@SiO_2_. Figure S5: Cycling performance of LiFePO_4_|P(VDF-HFP)@SiO_2_-GE|Li cell at 1 C. Figure S6: Nyquist plots for the LiFePO_4_|P(VDF-HFP)@SiO_2_-GE|Li cell acquired in the frequency range of 10 Hz to 100 KHz before and after 1000 cycles at the current density of 1 C. Figure S7: The voltage profile of the first three cycles of F-C@SiO_2_|P(VDF-HFP)@SiO_2_-GE|Li cell at current density of 0.2 A g^−1^. Table S1: Comparison of ionic conductivity (σ), electrolyte adsorption rate, capacity and capacity retention ratio of different gel electrolytes.
