# Supplementary material for: Fabrication of Composite Gel Electrolyte and F-Doping Carbon/Silica Anode from Electro-Spun P(VDF-HFP)/Silica Composite Nanofiber Film for Advanced Lithium-Ion Batteries
Source: Molecules. 2023 Jul 10;28(14):5304. doi: 10.3390/molecules28145304 (PMC10385190; doi:10.3390/molecules28145304)
Supplement: Supplementary file 1 [file molecules-28-05304-s001.zip › molecules-2491541-supplementary.pdf]

Fabrication of Composite Gel Electrolyte and F-Doping Carbon/Silica Anode from Electro-Spun P(VDF-HFP)/Silica Composite Nanofiber Film for Advanced Lithium-Ion Batteries

Caiyuan Liu, Xin Fang, Hui Peng, Yi Li * and Yonggang Yang

Jiangsu Key Laboratory of Advanced Functional Polymer Design and Application, Department of Polymer Science and Engineering, College of Chemistry, Chemical Engineering and Materials Science, Soochow University, Suzhou 215123, China

* Correspondence: liyi@suda.edu.cn; Tel.: +86-512-6588-2052

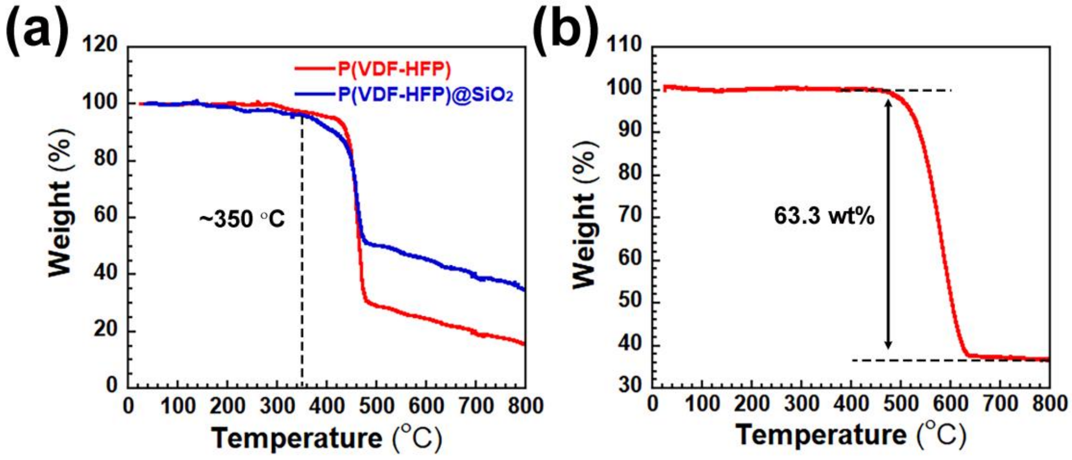

Figure S1. TGA curve of (a) P(VDF-HFP)@SiO_2 and (b) F-C@SiO_2.

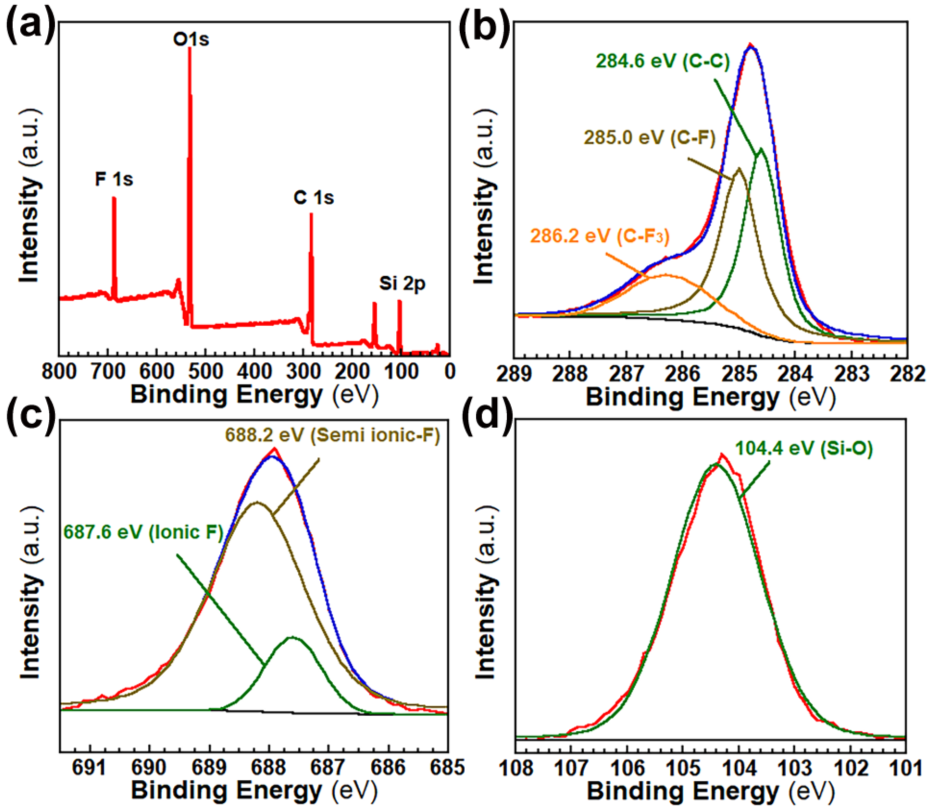

Figure S2. The XPS curve of F-C@SiO_2 composite nanofibers: (a) full spectrum, (b) C 1s, (c) F 1s, (d) Si 2p orbital high-resolution spectrum.

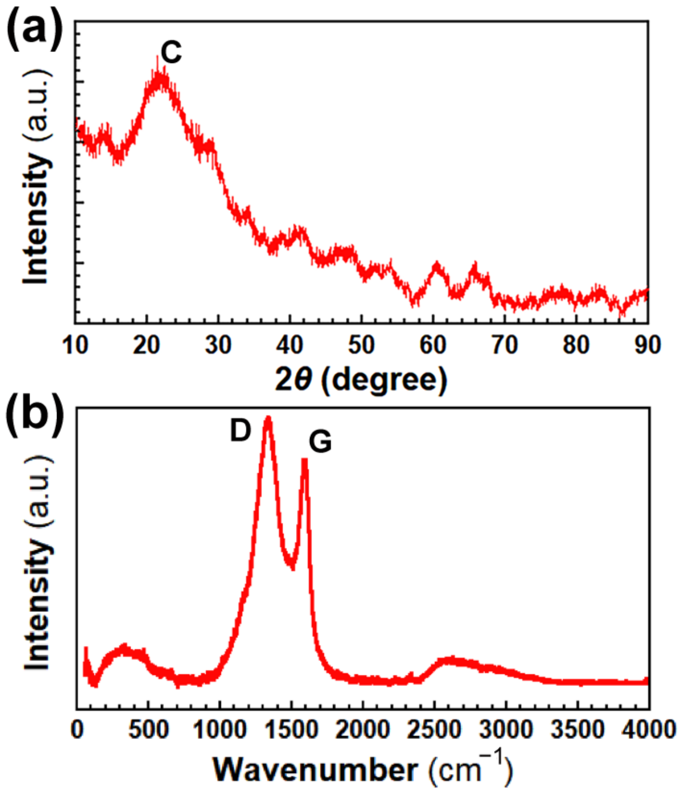

Figure S3. (a) WAXRD pattern and (b) Raman spectrum of F-C@SiO_2.

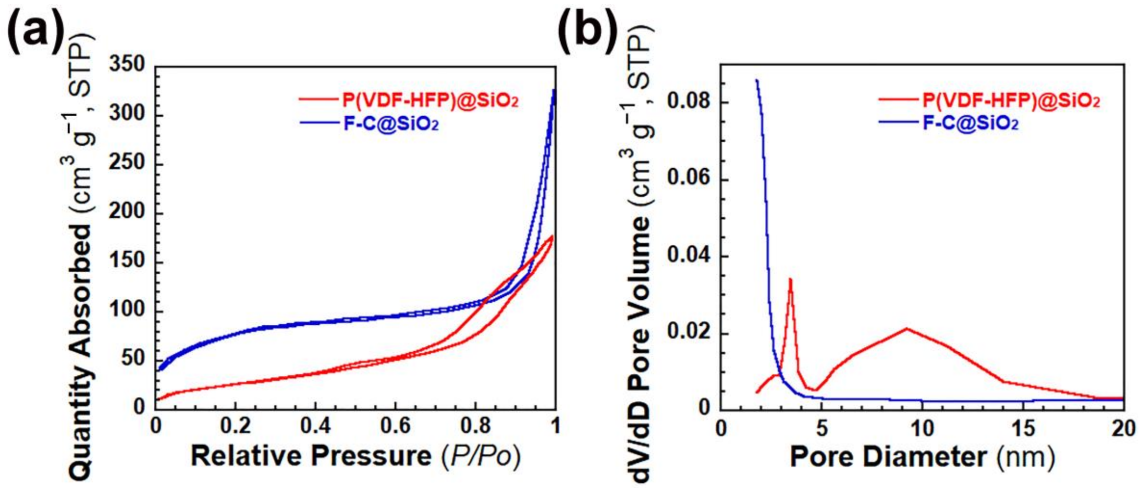

Figure S4. (a) Nitrogen sorption isotherms and (b) BJH pore size distribution plot calculated from the adsorption branch of P(VDF-HFP)@SiO_2 and F-C@SiO_2.

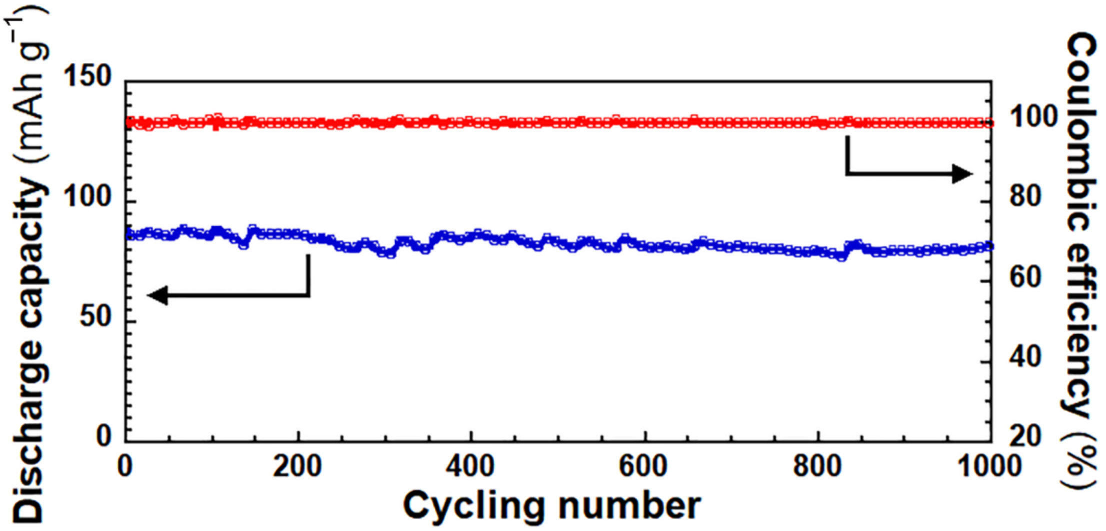

Figure S5. Cycling performance of $LiFePO_4$|P(VDF-HFP)@SiO_2-GE|Li cell at 1C.

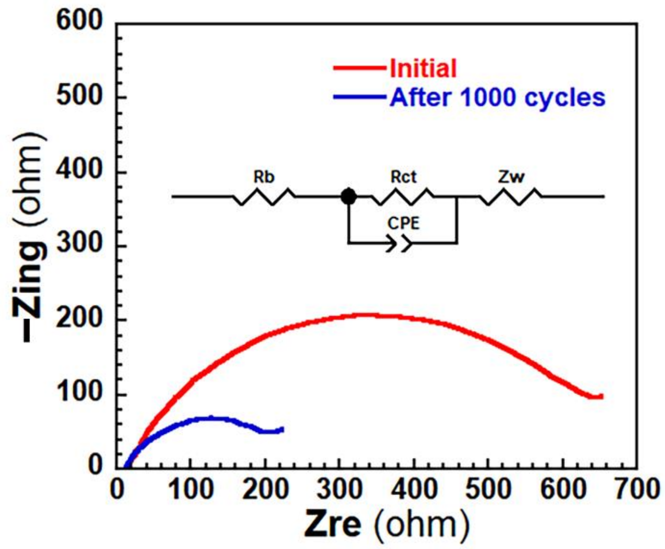

Figure S6. Nyquist plots for the $LiFePO_4$|P(VDF-HFP)@SiO_2-GE|Li battery acquired in the frequency range of 10 Hz to 100 KHz before and after 1000 cycles at the current density of 1C.

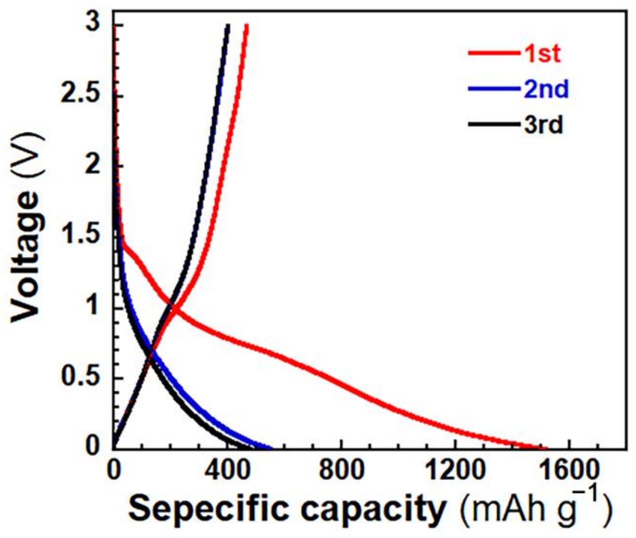

Figure S7. The voltage profile of the first three cycles of F-C@SiO_2|P(VDF-HFP)@ SiO_2-GE|Li cell at current density of 0.2 A g^{-1}.

Table S1. Comparison of ionic conductivity (σ), electrolyte adsorption rate, capacity and capacity retention ratio of different gel electrolytes.

Gel electrolyte	σ (mS cm^{-1})	Electrolyte Uptake (%)	Capacity (mAh g^{-1})	Capacity retention ratio (%)	Ref.
P(VDF-HFP)	1.42	420	133.2 (1C)	95.9 (500 cycles, 1C)	[1]
P(VDF-HFP)/ LLZA	2.25	330	160 (0.1C)	87.5 (100 cycles, 0.1C)	[2]
PMIA@P(VDF-HFP)/Al_2O_3	0.908	445	138.2 (0.1C)	95.6 (600 cycles, 1C)	[3]
P(VDF-HFP)-SiO_2@PPC	1.05	460	167 (1C)	94 (100 cycles, 1C)	[4]
P(VDF-HFP) @SiO_2	2.6	1000	134 (0.1C)	95 (1000 cycles, 1C)	This work

References

1. Wang, L.; Zhang, R.; Dai, H.; Yan, J.; Zhang, J.; Zhong, M.; Shen, W.; Guo, S. Reinforce the Adhesion of Gel Electrolyte to Electrode and the Interfacial Charge Transfer via In Situ Electrospinning the Polymeric Nanofiber Matrix. *Energy Technol.* **2021**, *9*, 2000865.

2. Karthik, K.; Din, M. M. U.; Jayabalan, A. D.; Murugan, R. Lithium garnet incorporated 3D electrospun fibrous membrane for high capacity lithium-metal batteries. *Mater. Today Energy* **2020**, *16*, 100389.

3. Wang,Lei.; Yan, J.; Zhang, R.; Li, Y.; Shen, W.; Zhang, J.; Zhong, M.; Guo, S. Core−Shell PMIA@PVdF-HFP/Al_2O_3 Nanofiber Mats In Situ Coaxial Electrospun on $LiFePO_4$ Electrode as Matrices for Gel Electrolytes. *ACS Appl. Mater. Inter.* **2021**, *13*, 9875−9884.

4. Song, X.; Qi, W.; Zhang, H.; Wang, G. Construction of core-shell nanofiber membrane with enhanced interface compatibility for lithium-metal battery. *Solid State Ionics* **2020**, *347*, 115266.
